# Population Density, Poor Sanitation, and Enteric Infections in Nueva Santa Rosa, Guatemala

**DOI:** 10.4269/ajtmh.15-0555

**Published:** 2016-04-06

**Authors:** Claudia Jarquin, Benjamin F. Arnold, Fredy Muñoz, Beatriz Lopez, Victoria M. Cuéllar, Andrew Thornton, Jaymin Patel, Lisette Reyes, Sharon L. Roy, Joe P. Bryan, John P. McCracken, John M. Colford

**Affiliations:** Center for Health Studies, Universidad del Valle de Guatemala, Guatemala City, Guatemala; Division of Epidemiology, School of Public Health, University of California, Berkeley, Berkeley, California; Waterborne Diseases Prevention Branch, Division of Foodborne, Waterborne, and Environmental Diseases, U.S. Centers for Disease Control and Prevention, Atlanta, Georgia; Department of Epidemiology, Gillings School of Global Public Health, University of North Carolina at Chapel Hill, Chapel Hill, North Carolina; Ministry of Public Health and Social Assistance, Cuilapa Health Area, Guatemala City, Guatemala; International Emerging Infections Program, Division of Global Health Protection, Centers for Disease Control and Prevention Central American Regional Office, Guatemala City, Guatemala

## Abstract

Poor sanitation could pose greater risk for enteric pathogen transmission at higher human population densities because of greater potential for pathogens to infect new hosts through environmentally mediated and person-to-person transmission. We hypothesized that incidence and prevalence of diarrhea, enteric protozoans, and soil-transmitted helminth infections would be higher in high-population-density areas compared with low-population-density areas, and that poor sanitation would pose greater risk for these enteric infections at high density compared with low density. We tested our hypotheses using 6 years of clinic-based diarrhea surveillance (2007–2013) including 4,360 geolocated diarrhea cases tested for 13 pathogens and a 2010 cross-sectional survey that measured environmental exposures from 204 households (920 people) and tested 701 stool specimens for enteric parasites. We found that population density was not a key determinant of enteric infection nor a strong effect modifier of risk posed by poor household sanitation in this setting.

## Introduction

Most enteric pathogens are spread by fecal–oral transmission that is facilitated by the close contact of people either through direct, person-to-person transmission or through indirect, environmentally mediated transmission.^1^ Historically, high densities of people living in congested urban areas suffered from intense enteric disease burdens until environmentally mediated transmission was interrupted through municipal water treatment and improved sewerage.^2^–^4^ Effective sanitation that ensures the safe disposal of human feces and prevents contact with future human hosts is a primary prevention barrier for enteric pathogen transmission,^5^ and studies show that improved sewerage reduces diarrheal disease and enteric parasite infections.^6^ Improved sanitation conditions have been generally associated with reduced diarrhea^7^ and soil-transmitted helminth (STH) infections,^8^ but three recent sanitation intervention studies in rural India found no reductions in diarrhea or STH infections despite large improvements in latrine coverage^9^–^11^—one of which found no evidence for effect modification of sanitation improvements by local population density.^11^ A limitation of the sanitation intervention studies was imperfect compliance, which could have underestimated the benefits of universal sanitation, but heterogeneity in the risk posed by poor sanitation has been predicted by theory due to the complexity of enteric pathogen transmission and the importance of environmental context to transmission.^1^,^12^

Population density may be one important source of heterogeneity, and if so, enteric infection studies designed explicitly around questions of population density and its relationship to poor sanitation will help guide future intervention programs. Yet, we are aware of few empirical studies of the relationship between population density and enteric infection risk.^13^–^15^ Most studies have found higher enteric infection risk at higher population densities,^13^,^15^ but not in every case—for example, STH reinfection rates were highest in low-density areas of a study in rural Panama.^14^

The objectives of this study were to compare enteric infection rates at high- and low-population densities in a Guatemalan municipality and to determine whether poor sanitation conditions pose a greater risk at high-population density compared with low-population density. Due to the greater opportunity for individuals to come into contact with fecal pathogens through contact with their environment or with other people at higher population densities, we hypothesized that enteric infections would be more common in high-density areas compared with low-density areas and that poor sanitation would pose a greater risk for enteric pathogen infection at higher population density. Since poor sanitation theoretically contributes most to environmentally mediated enteric pathogen transmission, we would expect it to pose a greater risk for environmentally mediated pathogens than for those spread predominantly through person-to-person contact.^16^

## Materials and Methods

### Setting and study overview.

Nueva Santa Rosa is a municipality located southeast of Guatemala City in the department of Santa Rosa. Its population in 2011 was approximately 33,000, and 80% of people live in rural areas, whereas 20% live in urban areas. Population density ranges from extremely low (dispersed rural farms) to moderate (regional urban centers). Unlike many regions of Guatemala, just 3% of Santa Rosa's population is indigenous, and 91% speak Spanish as their first language.

The study used two complementary data sources. The first source was an ongoing diarrhea surveillance system (*Vigilancia Integrada Comunitaria* [VICo]) in the municipality that tested stool specimens from individuals who presented to six sentinel clinics with diarrhea symptoms^17^–^19^ from 2007 to 2013. The surveillance data enabled us to estimate medically attended diarrhea rates in high- and low-density areas of the municipality. The second source was a 2010 cross-sectional survey in the municipality, which collected detailed information about household water, sanitation, and hygiene conditions, and tested stool specimens for enteric parasites. The cross-sectional survey provided information about enteric infection prevalence at the household level and enabled us to test whether infections associated with poor sanitation differed by high- and low-population density. Institutional review boards at the Centers for Disease Control and Prevention (cross-sectional survey) and the Universidad del Valle de Guatemala (VICo surveillance and cross-sectional survey) reviewed and approved study protocol amendments for this analysis, and all participants provided informed consent.

### Cross-sectional survey design and outcome measurement.

The cross-sectional survey was conducted in August–September 2010. The study drew a simple random sample of 387 roofs from all 10,770 possibly inhabited roofs (16–150 m^2^) using global positioning system coordinates identified in aerial imagery. From these 387 roofs, the final survey enrolled 204 households and 920 individuals. The reasons for 183 roofs not being enrolled were the following: structure not found by interviewers (*N* = 12, 3%), structure not being used for living purposes (*N* = 65, 17%), structure uninhabited at the time of the visit by field workers (*N* = 58, 15%), no adult was present at the time of the visit to provide consent for participation (*N* = 2, 0.5%), and not providing consent to participate in the study (*N* = 44, 11%). Of the remaining 211 consenting households, seven participated in the survey but their data were removed from analysis because of data cleaning issues (e.g., missing data, inconsistencies of answers that could not be resolved). Interviewers recorded observations of sanitation facility condition and use, observed feces in the household environments, and reported child feces disposal practices. Interviewers collected information about housing conditions, assets, and environmental conditions, including: water sources, water treatment practices, water quality, observations of the primary handwashing locations, and animal presence around the home.

Interviewers asked the primary respondent, usually an adult female (76%), about diarrhea symptoms (≥ 3 loose or watery stools in 24 hours) in the past 7 days for each household member.^20^–^22^ We analyzed the results of stool parasite testing from 701 (78%) of 904 study participants known to be > 12 months of age. Field staff collected stool specimens from individuals > 12 months old who provided consent, preserved them in formalin, and tested them for the presence of STH (*Ascaris lumbricoides*, *Trichuris trichiura*, hookworm [*Ancylostoma* or *Necator*]), protozoans (*Giardia lamblia*, *Entamoeba histolytica*, *Entamoeba coli*, *Blastocystis hominis*), and tapeworms (*Hymenolepis nana*, *Hymenolepis diminuta*) using the fecal parasite concentrator method (Midi Parasep^®^, DiaSys DYS001; DiaSys, Berkshire, England).

### VICo surveillance design and outcome measurement.

The VICo health facility–based surveillance system collected stool specimens between September 2007 and December 2013 to monitor infectious causes of diarrhea.^17^–^19^ The program collected stool specimens from all individuals who presented at care facilities in the urban center in Nueva Santa Rosa (*N* = 2,557) and five rural health posts (*N* = 1,803) in the municipality who met the diarrhea case definition: ≥ 3 loose or liquid stools in a 24-hour period with onset in the past 7 days. We excluded diarrhea cases from the regional hospital in Cuilapa (*N* = 1,104) because the hospital receives patients from the entire department. Cases originating in the Nueva Santa Rosa municipality typically present first to the municipal clinics before referral (for severe cases) to the Cuilapa hospital. Such referrals were not tracked in the surveillance system and excluding cases from the Cuilapa hospital ensured that we did not double count cases originating in Nueva Santa Rosa. Each stool sample (or rectal swab if whole stool was not possible) included case demographic characteristics and socioeconomic characteristics; VICo collected few environmental and household exposure characteristics related to enteric infections, except for drinking water source and floor material.

The details of VICo specimen collection and testing have been previously described^17^–^19^; the Supplemental Text includes additional details. Samples were tested for STH (*A. lumbricoides*, *T. trichiura*, hookworm [*Ancylostoma* or *Necator*]), protozoan parasites (*G. lamblia*, *E. histolytica*, *E. coli*, *B. hominis*), and tapeworms (*H. nana*, *H. diminuta*) by direct smear microscopic examination^23^; for bacteria (*Salmonella* spp., *Shigella* spp., *Campylobacter* spp.) by direct culture^24^; for *Escherichia coli* pathotypes (enterotoxigenic *E. coli*, enteropathogenic *E. coli*, and Shiga toxin-producing *E. coli*) using conventional polymerase chain reaction^25^; for rotavirus (group A) by using a commercial qualitative enzyme immunoassay (IDEIA Rotavirus test kits; Oxoid Ltd., Ely, United Kingdom)^17^; and for norovirus (genogroups I and II) using a standard monoplex quantitative reverse transcription polymerase chain reaction.^18^,^26^ Supplemental Table 1 summarizes the number of VICo samples and pathogen-specific test results.

### Population density estimation.

For each household in the cross-sectional survey, we identified the number of roofs within a 50-m radius. We chose a 50-m radius to be consistent with recent enteric infection sanitation studies conducted in urban and rural environments,^11^,^27^ and because prior studies suggest 50 m is a relevant scale for environmental enteric pathogen transmission.^28^,^29^ We estimated each study household's population density in persons per square kilometer by multiplying the number of roofs within a 50-m radius by the number of people per roof in the study household. Of 204 households in the cross-sectional study, 75 (37%) had multiple roofs and the number of roofs associated with each household varied from one to six. Due to the relatively small number of households, we reduced the continuous population density estimates to a dichotomous classification of “high density” or “low density” using a prespecified method that allowed us to select a cut point that had the best agreement with a blinded investigator consensus classification of high and low density (Supplemental Text). The cut point corresponded to 5,348 persons/km^2^, which was the 74th percentile of the distribution.

Each diarrhea case identified through VICo surveillance had a recorded home village or populated place (e.g., neighborhood). We matched each case to the official list of populated places from Guatemala's Instituto Nacional de Estadística (INE), and we excluded four cases that we could not match to a location. The remaining 4,360 cases originated from 31 locations in the municipality. We calculated village- and age-specific population estimates for 2010 (midpoint of the 2007–2013 surveillance) by increasing location-specific 2002 census populations by INE municipal-level growth rates through 2010.^17^ To estimate a location's population density, we divided its 2010 population by its area and we calculated areas with a village boundary dataset from Guatemala's Ministry of Agriculture (median location area = 0.9 km^2^, maximum = 5 km^2^; median location density = 754 persons/km^2^, maximum = 2,579 persons/km^2^). We converted the continuous population density measures into dichotomous groups by classifying households above the 75th percentile of the distribution (> 1,005 persons/km^2^) as “high density.” We used a 75th percentile cutoff to maintain consistency with our high-density definition from the cross-sectional survey.

### Sanitation definition and classification.

In the cross-sectional survey we defined a household's sanitation as “improved” if: 1) it had a toilet that met the World Health Organization/United Nations Children's Fund Joint Monitoring Program (WHO/UNICEF JMP) criteria^30^ based on staff observation, 2) the toilet was located on the premises and not shared with the public (reported information), 3) the toilet was cleaned in the past 4 weeks (reported information), and 4) the last time the youngest child in the household passed stool, it was disposed in a toilet (reported information). Households who did not meet all four conditions were classified as having poor sanitation. The current WHO/UNICEF JMP definition of improved sanitation focuses only on toilet hardware and does not incorporate information about whether and how the toilet facilities are used. We included measures of toilet use, which is required to reduce pathogenic excreta in the environment. We attempted to characterize neighborhood sanitation in the study area, but we deemed the algorithm's prediction error too high for the exposure to be meaningful (Supplemental Information).

### Statistical analysis.

Using the surveillance data, we calculated crude incidence rates by dividing incident cases by the person-time at risk in the municipality, which we estimated using the surveillance dates and 2010 population estimates described above.^19^ We compared medically attended diarrhea incidence rates between high- and low-density locations using an incidence rate ratio (IRR). We calculated adjusted IRRs using a pooled Mantel–Haenszel estimator of the IRR,^31^ stratified by distance to the closest surveillance site (< 1 km versus ≥ 1 km measured by the centroid of the populated place) and by age (< 5 years old versus ≥ 5 years old). We stratified the distance at 1 km because it was a natural break in the distance distribution. We estimated the IRR for all-cause diarrhea and for pathogen-specific causes of diarrhea for pathogens associated with ≥ 5% of cases to ensure sufficient sample sizes (*Shigella* spp., *Campylobacter* spp., pathogenic *E. coli*, rotavirus, norovirus).

**Figure 1.**
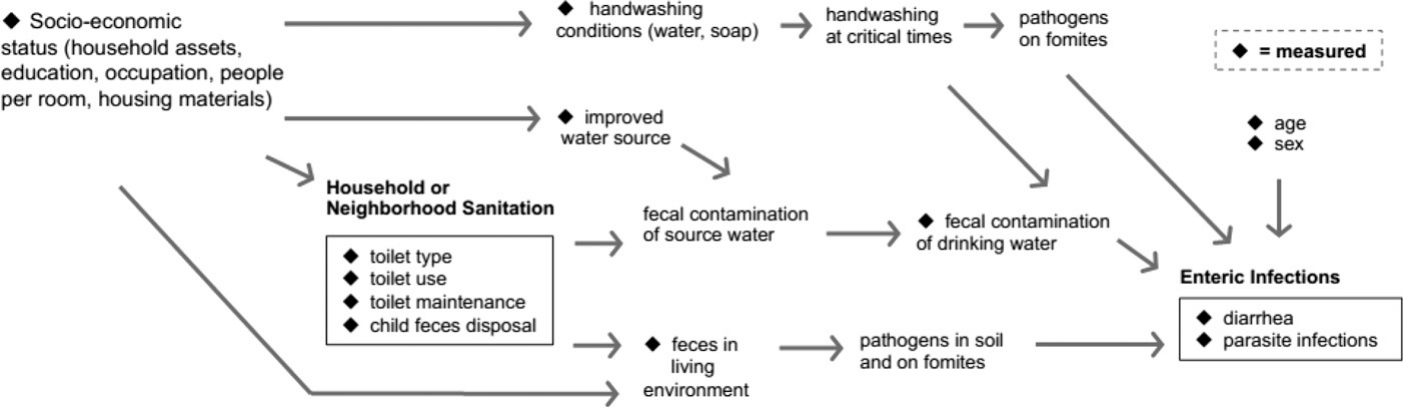
Hypothesized causal relationship between poor sanitation and enteric infections, including major potential confounders and intermediate outcomes.

We compared the prevalence of each enteric infection between high-density areas and low-density areas in the cross-sectional survey. We examined whether household sanitation was differentially associated with enteric infection prevalence by estimating the association between poor sanitation conditions and enteric infection prevalence separately in low- and high-density households, controlling for possible confounding factors informed by our causal model (Figure 1

). We estimated the unadjusted and adjusted prevalence ratio (PR) associated with high-density areas, as well as the PR associated with poor sanitation, stratified by high- and low-population density. We examined whether the association between enteric infections and poor sanitation was modified by population density on the additive scale because we were interested in whether the effect of poor sanitation would be greater in high-density compared with low-density households with the aim of targeting future interventions to specific populations.^32^ We quantified effect modification with the relative excess risk due to interaction (RERI), which assesses whether the effect of the two exposures together exceeds the sum of their effects when considered separately (a RERI value > 0 indicates positive effect modification).^32^ The Supplemental Text includes minimum detectable effect calculations, statistical analysis details, and some exploratory analyses of the geographic distribution of infection.

## Results

### Participant characteristics.

Grouping VICo cases into those among people living in low- and high-density areas resulted in 1,650 diarrhea cases (low density) and 2,710 cases (high density). In the cross-sectional survey, the classification of households into low- and high-density areas resulted in 57 high-density households (292 people) and 147 low-density households (628 people). Characteristics of the cross-sectional survey households were similar to households of diarrhea cases detected through VICo surveillance, except for participant age—young children accounted for the majority of VICo cases (Table 1).

### Medically attended diarrhea incidence rates at high and low densities.

**Figure 2.**
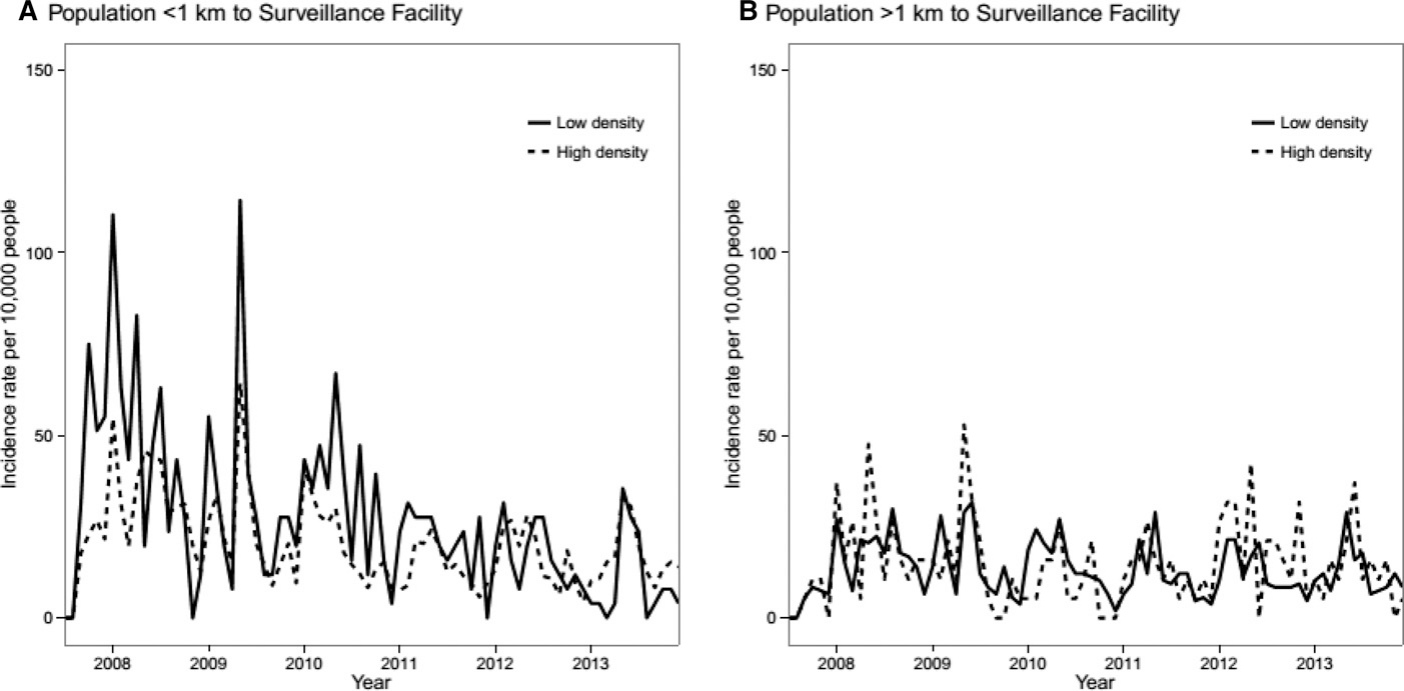
Incidence of medically attended diarrhea in VICo (*Vigilancia Integrada Comunitaria*) surveillance sites over time, stratified by distance to a surveillance site and by high- and low-population density. High-density areas were defined as those in the top 25th percentile of the population density distribution. Nueva Santa Rosa, Guatemala (2007–2013).

Distance to the surveillance facility was a strong confounder of the association between population density and diarrhea because it was strongly, positively associated with both population density and the probability of reporting to a surveillance site. For this reason, our analysis focused on adjusted IRR estimates that stratified by distance and age. Diarrhea incidence was lower in high-density areas versus low-density areas after adjusting for distance to surveillance site and age (IRR = 0.85, 95% confidence interval [CI] = 0.79, 0.92) (Figure 2

, Table 2, and Supplemental Table 2 includes stratified IRRs). Pathogen-specific adjusted incidence rates were higher in high-density areas for *Shigella* spp. (IRR = 1.38, 95% CI = 1.00, 1.90) and rotavirus (IRR = 1.56, 95% CI = 1.03, 2.36).

### Enteric infection prevalence at high and low densities.

We detected no statistically significant differences in prevalence between high- and low-density households in the cross-sectional survey (Table 3). Diarrhea prevalence was similar in the two groups (PR = 1.05, 95% CI = 0.51, 1.86). There were no detected infections of *E. histolytica* in the study population, and prevalence of *T. trichiura* hookworm, *Hymenolepis nana*, *H. diminuta*, and *B. hominis* was < 2%. Among STH infections, *A. lumbricoides* was most prevalent but we observed similar prevalence in households in high- and low-density areas (10.0% versus 8.3%; PR = 1.22, 95% CI = 0.50, 2.50). Although not statistically different, protozoan parasite prevalence tended to be lower in high-density households: *G. lamblia* prevalence was 4.8% in high-density households compared with 7.8% in low-density households (PR = 0.61, 95% CI = 0.26, 1.08), and *E. coli* prevalence was 24.5% in high-density households compared with 33.3% in low-density households (PR = 0.74, 95% CI = 0.50, 1.01).

### Association between poor sanitation and enteric infections.

Poor household sanitation was not associated with diarrhea at either low density (PR = 1.04, 95% CI = 0.51, 2.17) or high density (PR = 0.68, 95% CI = 0.25, 1.79), nor was it consistently associated with *G. lamblia* infection (Table 4). In unadjusted analyses, the association between poor sanitation and *A. lumbricoides* infection was stronger in high-density areas (PR = 3.49, 95% CI = 1.03, 22.81) compared with low-density areas (PR = 1.63, 95% CI = 0.59, 4.83), with some evidence for effect modification (RERI = 1.04, 95% CI = −1.45, 3.72). However, after adjusting for potentially confounding covariates, there was no evidence for an association between poor sanitation conditions and *A. lumbricoides* infection and no evidence for effect modification by population density (Table 4). Due to large amounts of spatial overlap between poverty, poor sanitation, and high density, it was difficult to individually separate their effects in this population (Supplemental Figures 1–2).

## Discussion

In this setting, and within the limitations of this study, we found that higher population density on its own did not increase the risk of environmentally mediated enteric infections (e.g., STH, *Campylobacter* spp., pathogenic *E. coli*), but we did observe some evidence for higher adjusted incidence rates of medically attended diarrhea attributed to *Shigella* spp. and rotavirus in higher density areas—both pathogens are most likely dominated by person-to-person transmission.^33^ In contrast, we observed lower adjusted rates of all-cause, medically attended diarrhea in higher density areas (Table 2). There was a trend toward lower prevalence of *G. lamblia* and *E. coli* infection within high-density households, but the differences were not statistically significant (Table 3). *Entamoeba coli* is not typically considered pathogenic,^34^ but it is a measure of fecal–oral transmission, particularly through food and waterborne pathways. We observed no consistent pattern of population density acting as an effect modifier of enteric infection risk posed by poor sanitation. This is important because it suggests that interventions to reduce enteric infections, such as improved sanitation, should not necessarily focus efforts solely on high-density populations.

Population density is a complex exposure and readers should interpret our findings in the context of the study's methods and setting. There are three main points to consider. First, the study included households across a wide range of population densities (Supplemental Figure 4), but the range of density we studied was lower than in large city centers and urban slum environments—for example, the population density of slums in Dhaka, Bangladesh, is 205,415 people/km^2^ 35—10–100 times higher than densities observed in this study. If population density affects the relationship between poor sanitation and enteric infection risk in a nonlinear way, with stronger effect modification at highest densities—a pattern seen for infant mortality^36^—then the range of population density observed in this study may have been too low to adequately compare high- and low-density conditions with respect to their role in enteric pathogen transmission. A forthcoming intervention study at higher population density in Mozambique will contribute additional evidence to this question.^37^

Second, we reduced the continuous range of population densities into dichotomous groups of high and low density that corresponded to the 74th percentile of the density distribution. We dichotomized population density due to the relatively small study sample. We chose the cut point using a prespecified and repeatable method designed to reflect local conditions (Supplemental Text), but in larger studies it would be useful to look for associations and effect modification across a finer gradient of population density. When we stratified the medically attended diarrhea rates by quartiles of population density, we saw no clear dose–response relationship (Supplemental Table 3); the cross-sectional survey was sufficiently small that we felt we could not conduct a similar analysis.

Finally, we used two different approaches to estimate population density that arose from our different data sources. For the surveillance data, we estimated population density for populated places using their estimated boundaries and estimated population. The areas were small (median = 0.9 km^2^), but were larger than areas used to calculate 50-m point densities in the cross-sectional survey (area = 0.008 km^2^). Population densities calculated over larger areas will be lower because larger areas include more open space,^38^ so we chose to use a similar quantile of the distribution to define high and low density. Nevertheless, comparison of results across analyses of population density will only be perfectly comparable if they calculate population density using the same method. We chose a 50-m radius around study households to characterize point density to be comparable to previous sanitation studies,^11^,^27^ with the rationale that a 50-m spatial scale was most relevant for environmentally mediated pathogen transmission.^28^,^29^ The choice is not evidence based within this setting, however, and the relevant radius used for point density calculation could vary by environment.

This study had some limitations owing to the use of previously collected data to test our hypotheses. These limitations would not obtain in a prospective study designed de novo to test these hypotheses, and we hope that the limitations encountered in this analysis help guide future research. First, our stratified estimates using the surveillance data corrected for age and distance to the surveillance site, but it remains possible that there was residual confounding between high- and low-density areas that we could not adjust for in the analysis. Second, the cross-sectional survey that we used to test the joint relationship between population density and poor sanitation was relatively small (*N* = 701 stool specimens). The study had power to detect stratified PRs associated with poor sanitation of between 2.13 and 2.81, and a RERI of ≥ 2.25, but many of the associations estimated in this study were smaller than this (Table 4). This limited our ability to rule out smaller effects and underscores the importance of large sample sizes in effect modification studies.^39^ We found similar results when we repeated the analyses using a more sensitive, composite definition for STH infection—fecal parasite concentration + Kato-Katz in a validation subsample (Supplemental Table 4). Third, the cross-sectional analysis estimated population densities by applying the number of residents per roof in the study households to all roofs in a 50-m radius—a complete census would be more accurate. Fourth, we used a single exposure definition of “poor sanitation” in low- and high-density areas, which was consistent with estimating effects under a counterfactual causal model. Yet, if the features of household or neighborhood sanitation conditions that drive enteric pathogen transmission differ depending on population density, a single definition of poor sanitation across different densities could potentially lead to misclassification of exposure, and in turn, mask some heterogeneity between enteric infection risk associated with poor sanitation in low- versus high-density areas. Finally, the cross-sectional analysis focused on household-level sanitation conditions and not on neighborhood sanitation conditions. This approach implicitly assumes that household sanitation conditions are most relevant for enteric infection risk or that household conditions reflect the broader sanitation conditions in the relevant transmission area around a household. We could not test this assumption because we found that predictions of neighborhood sanitation conditions using spatial location were unreliable (Supplemental Information). A measurement approach that characterized sanitation conditions over the same area used to calculate population density would be the best approach for future studies because it would better characterize the joint exposures of poor sanitation and population density within a spatially relevant transmission area.

## Conclusion

In the rural and small urban areas of Nueva Santa Rosa, Guatemala, population density was not a major determinant of enteric pathogen transmission, nor did it act as a strong effect modifier of risk posed by poor household sanitation.

## Supplementary Material

Supplemental Datas.

## Figures and Tables

**Table 1:** Study population characteristics, stratified by population density* (Nueva Santa Rosa, Guatemala)

Characteristics	VICo cases, 2007–2013			NSR cross-sectional survey, 2010		
	Low density %	High density %	Total %	Low density %	High density %	Total %
Individual characteristics						
Age, years (median [IQR])	2 (1–8)	2 (1–9)	2 (1–9)	21 (11–44)	19 (8–35)	20 (10–41)
Female	44	46	45	47	44	46
Distance to health facility						
Distance traveled, kilometer (median [IQR])	2.9 (1.2–4.6)	0.3 (0.1–2.1)	0.6 (0.3–4.3)	NA		
Distance to health facility, % < 1 km	33	92	70			
Household head, patient/guardian† education						
Did not complete primary	73	66	69	70	67	69
Completed primary	15	16	16	19	16	18
Completed secondary or more	12	18	16	11	18	13
Household environment						
Persons per sleeping room (median [IQR])	NA			2.0 (1.5–3.0)	2.5 (1.7–3.3)	2.3 (1.5–3.0)
Soil floor	42	37	39	33	32	33
Electricity	84	87	86	93	91	92
Cooks with biofuel	73	57	63	71	56	67
Handwashing, water and soap present	NA			66	82	71
Primary drinking water source						
Private tap	54	37	43	39	21	34
Public tap	34	37	36	20	35	24
Bottled water	10	25	19	24	35	27
Other	2	1	2	17	9	15
Wealth index quartile						
Quartile 1 (poorest)	24	26	25	27	28	27
Quartile 2	32	29	30	24	23	24
Quartile 3	20	19	19	27	19	25
Quartile 4 (richest)	23	26	25	23	30	25
Sanitation conditions						
JMP-defined improved sanitation	NA			75	77	75
Toilet on premises, not shared with public				81	81	81
Toilet cleaned in past 4 weeks				85	93	87
Child's last stool disposed in toilet				70	60	67
Poor sanitation‡	NA			54	61	56

IQR = interquartile range; JMP = Joint Monitoring Program; NA = not applicable; NSR = Nueva Santa Rosa; VICo = *Vigilancia Integrada Comunitaria*.

* In VICo surveillance, high-density areas were defined as populated places in the top 25% of population density for the NSR municipality. In the NSR cross-sectional study, households in locations > 5,348 persons/km^2^ were classified as high density.

† In VICo surveillance, information on education level is obtained from the patient/guardian. In NSR cross-sectional study, information was obtained from the head of household.

‡ Households were classified as having poor sanitation if they did not have all of the four sanitation characteristics listed in the table.

**Table 2:** Episodes, incidence rates, and IRRs for medically attended diarrhea cases in high-density vs. low-density areas in Nueva Santa Rosa, Guatemala, VICo surveillance, 2007–2013

Outcome	Low density			High density			High vs. low density IRR (95% CI)	High vs. low density adjusted IRR (95% CI)†
	Episodes	Person years at risk	Rate*	Episodes	Person years at risk	Rate*		
Diarrhea (all cause)	1,650	83,713	197.10	2,710	111,177	243.76	1.24 (1.16, 1.32)	0.85 (0.79, 0.92)
Pathogen-specific diagnoses								
*Shigella* spp.	93	83,713	11.11	295	111,177	26.53	2.39 (1.89, 3.05)	1.38 (1.00, 1.90)
*Campylobacter* spp.	108	83,713	12.90	243	111,177	21.86	1.69 (1.35, 2.15)	1.09 (0.83, 1.44)
Pathogenic *Escherichia coli*	90	28,629	31.44	188	38,022	49.45	1.57 (1.22, 2.05)	1.08 (0.77, 1.50)
Norovirus	114	82,409	13.83	221	109,444	20.19	1.46 (1.16, 1.85)	0.86 (0.66, 1.12)
Rotavirus	75	83,278	9.01	210	110,599	18.99	2.11 (1.61, 2.78)	1.56 (1.03, 2.36)

CI = confidence interval; IRR = incidence rate ratio; VICo = *Vigilancia Integrada Comunitaria*. High-density areas were defined as populated places in the top 25% of population density for the Nueva Santa Rosa municipality.

* Incidence per 10,000 person-years.

† Mantel–Haenszel pooled IRR, adjusted for distance to closest health facility (< 1 km vs. ≥ 1 km) and age (< 5 years vs. ≥ 5 years).

**Table 3:** Enteric infection prevalence at low- and high-population density in the cross-sectional survey (Nueva Santa Rosa, Guatemala, 2010)

Outcome	Low density		High density		PR (95% CI)	Adjusted* PR (95% CI)
	*n*/*N*	%	*n*/*N*	%	High density	High density
Diarrhea	45/628	7.2	22/292	7.5	1.05 (0.51, 1.86)	0.89 (0.33, 3.00)
*Ascaris lumbricoides*	39/472	8.3	23/229	10.0	1.22 (0.50, 2.50)	1.07 (0.36, 2.55)
*Trichuris trichiura*	2/472	0.4	2/229	0.9		
Hookworm	2/472	0.4	0/229	0.0		
*Hymenolepis nana/Hymenolepis diminuta*	8/472	1.7	4/229	1.7		
*Giardia lamblia*	37/472	7.8	11/229	4.8	0.61 (0.26, 1.08)	0.53 (0.16, 1.48)
*Entamoeaba coli*	157/472	33.3	56/229	24.5	0.74 (0.50, 1.01)	0.78 (0.46, 1.22)
*Blastocystis hominis*	6/472	1.3	2/229	0.9		

CI = confidence interval; PR = prevalence ratio. Households in locations > 5,348 persons/km^2^ (74th percentile) were classified as high density.

* Adjusted for age, sex, household head education, people per room, floor material, electricity, cooks with biofuel, wealth index, handwashing location, and drinking water source.

**Table 4:** Enteric infections associated with poor sanitation, stratified by population density in the cross-sectional survey (Nueva Santa Rosa, Guatemala, 2010)

Outcome population density	Improved sanitation		Poor sanitation		Relative risk of poor sanitation stratified by density		Adjusted* relative risk of poor sanitation stratified by density	
	*n*/*N*	%	*n*/*N*	%	PR (95% CI)	RERI (95% CI)	PR (95% CI)	RERI (95% CI)
Diarrhea								
Low density	20/285	7.0	25/343	7.3	1.04 (0.51, 2.17)		0.95 (0.57, 1.78)	
High density	10/106	9.4	12/186	6.5	0.68 (0.25, 1.79)	−0.46 (−2.56, 0.67)	0.94 (0.58, 1.64)	−0.01 (−0.76, 0.54)
*Ascaris lumbricoides*								
Low density	13/212	6.1	26/260	10.0	1.63 (0.59, 4.83)		1.71 (0.61, 2.75)	
High density	4/97	4.1	19/132	14.4	3.49 (1.03, 22.81)	1.04 (−1.45, 3.72)	1.33 (0.42, 3.44)	−0.48 (−0.95, 0.57)
*Giardia lamblia*								
Low density	18/212	8.5	19/260	7.3	0.86 (0.46, 1.60)		0.64 (0.23, 1.10)	
High density	4/97	4.1	7/132	5.3	1.29 (0.35, 8.20)	0.28 (−0.73, 0.97)	0.60 (0.23, 1.10)	−0.06 (−0.56, 0.41)
*Entamoeba coli*								
Low density	60/212	28.3	97/260	37.3	1.32 (0.96, 1.86)		0.85 (0.56, 1.09)	
High density	18/97	18.6	38/132	28.8	1.55 (0.88, 3.08)	0.04 (−0.64, 0.60)	0.70 (0.46, 0.99)	−0.14 (−0.34, 0.12)

CI = confidence interval; PR = prevalence ratio; RERI = relative excess risk due to interaction = PR_11_ − PR_10_ − PR_01_ + 1. Households in locations > 5,348 persons/km^2^ (74th percentile) were classified as high density.

* Adjusted for age, sex, household head education, people per room, cooks with biofuel, wealth index, handwashing location, and drinking water source.
