# Supplementary material for: Population Density, Poor Sanitation, and Enteric Infections in Nueva Santa Rosa, Guatemala
Source: Am J Trop Med Hyg. 2016 Apr 6;94(4):912–9. doi: 10.4269/ajtmh.15-0555 (PMC4824239; doi:10.4269/ajtmh.15-0555)
Supplement: Supplementary file 1 [file SD11.pdf]

SUPPLEMENTAL INFORMATION

VICo surveillance stool specimen collection and testing. Clinic staff collected a stool specimen from enrolled individuals (whole stool or rectal swab if the former was not possible). The details of specimen collection and testing have been previously described.[17–19] Stool samples were stored at 4°C (rectal swabs in Cary–Blair media) and transported in temperature-monitored containers (4°C) within 24 hours of collection to the laboratory at the Cuilapa Regional Hospital for initial analysis. Samples were tested for the presence of soil-transmitted helminth (STH) infections (*Ascaris lumbricoides*, *Trichuris trichiura*, hookworm [*Ancylostoma* or *Necator*]), protozoan parasites (*Giardia lamblia*, *Entamoeba histolytica*, *Entamoeba coli*, *Blastocystis hominis*), and tapeworms (*Hymenolepis nana*, *Hymenolepis diminuta*) by direct smear microscopic examination[23]; for bacteria (*Salmonella* spp., *Shigella* spp., *Campylobacter* spp.) by direct culture[24]; for *Escherichia coli* pathotypes (enterotoxigenic *E. coli*, enteropathogenic *E. coli*, and Shiga toxin-producing *E. coli*) using conventional polymerase chain reaction[25]; for rotavirus (group A) by using a commercial qualitative enzyme immunoassay (IDEIA Rotavirus test kits; Oxoid Ltd., Ely, United Kingdom)[17]; and for norovirus (genogroups I and II) using a standard monoplex quantitative reverse transcription polymerase chain reaction.[18,26] The laboratory at the Universidad del Valle de Guatemala performed quality control assessments for all assays.

Selecting a dichotomous cut point for population density in the cross-sectional survey. We selected a systematic sample of 51 households (25% of 204) across the range of observed densities. An analyst prepared standardized aerial images of 100 × 100 m centered on each sampled household; images were blinded and did not include any identifying information about estimated density. Three independent investigators from the United States and Guatemala (BFA, CJ, JMC) then classified each household image into "high-" or "low-"density groups based on a qualitative assessment of the aerial images, with no restriction other than there were two classes of density. We derived an investigator consensus classification for each of the 51 sampled households by majority vote across the three reviewers. Primary reviewer agreement (BFA, CJ) was 94% (48/51), and there was unanimous classification of images for 69% (35/51). We used the ROCR package in R to calculate agreement between every possible density cut point in the subsample using the investigator consensus classification as the gold standard.[1] We used the cut point that maximized agreement (5,348 persons/km^2; 74th percentile of the distribution; average classification accuracy 85%) as the population density cut point for high and low density. Supplemental Figures 4 and 5 summarize the population density distribution and illustrate the average accuracy for different cut points.

Attempt to characterize neighborhood sanitation. We attempted to include sanitation measures at the neighborhood level (defined as a radius of 50 m around each study household), based on the sanitation information available for the random sample of 204 households who were surveyed among the 10,770 roofs identified in aerial imagery of the Nueva Santa Rosa municipality. We used a k-nearest neighbor algorithm[2] to estimate sanitation conditions for all 10,770 roofs in the community, assuming that sanitation conditions follow high levels of spatial correlation. The algorithm identified the k-nearest neighbors from the 204 households in the survey based on Euclidean distance, using majority voting to classify the sanitation conditions of the living structure. Five-fold cross-validation was used to select k that minimized the training set classification error in the 204 households. The cross-validated classification error for predicted sanitation conditions in the 204 study households was > 38% for all values of k, which exceeded our prespecified 20% error rate. Thus, we felt there was insufficient information in the data to accurately predict sanitation conditions for roofs in the study region based only on geographic location, which led us to limit the analysis to household sanitation conditions.

Statistical analysis details. Our parameter of interest for the association between enteric infections and the independent and combined exposures of population density and sanitation was the prevalence ratio (PR). The PR associated with poor sanitation ($A = 1$) within each stratum of population density ($D = d$) for outcome Y is:

$$\mathrm{PR} = E_W[P(Y|A=1, D=d, W)/P(Y|A=0, D=d, W)] \quad (1)$$

The marginal PR is averaged over covariates W. We examined whether the association between enteric infections and poor sanitation was modified by population density on the additive scale because we were interested in whether the effect of poor sanitation would be greater in high-density compared with low-density households with the aim of targeting future interventions to specific populations.[32] We quantified effect modification with the relative excess risk due to interaction (RERI), which assesses whether the effect of the two exposures together exceeds the sum of their effects when considered separately.[32] For the prevalence of an outcome under two dichotomous conditions (p_{AD}), the RERI is:

$$\begin{aligned}\mathrm{RERI} &= (p_{11}/p_{00}) - (p_{10}/p_{00}) - (p_{01}/p_{00}) + (p_{00}/p_{00}) \\ &= PR_{11} - PR_{10} - PR_{01} + 1 \quad (2)\end{aligned}$$

A RERI value > 0 indicates positive effect modification. Since this analysis relied on existing data, for our second objective (to determine whether poor sanitation poses a greater risk at high-population density compared with low-population density) we calculated the minimum detectable effect for the stratified PRs and the RERI associated with poor sanitation given the size of the study, the empirical distribution of poor sanitation by high and low density, and assuming 8% outcome prevalence in the improved sanitation group. The study was sufficiently large to detect a PR associated with poor sanitation of 2.13 (low density) and 2.81 (high density) with 80% power and a two-sided alpha of 5%. Using a simulation-based approach,[3,4] and assuming a PR = 2 associated with poor sanitation in the low-density stratum, we estimated that the minimum detectable RERI given the design was 2.25.

We computed adjusted estimates using targeted minimum loss–based estimation, which is a double-robust approach to adjust for covariates (W).[5] We used a data-adaptive ensemble machine learning algorithm[6] to flexibly control for covariates in all adjusted analyses; the algorithm included the following model selection approaches: main effects log-linear regression, stepwise Akaike Information Criterion,[7] generalized additive models,[8] and glmnet (lasso) regression.[9] We considered the following covariates: age, sex, household head education, people per room, biofuel use, wealth index quartile, handwashing location within 10 m of the toilet stocked with water and soap,

and drinking water supply. The wealth index was the first principal component from a principal components analysis[10] using the following household assets and income variables: refrigerator, computer, radio, clothes washer, clothes dryer, car/truck, television, telephone, microwave, watch, bicycle, motorcycle/scooter, and reported household income. The wealth index provides a relative measure of wealth within the study population. We selected covariates based on our hypothesized causal model (Figure 1)[31] to block any backdoor paths between sanitation conditions and enteric infections. We calculated percentile 95% confidence intervals for all parameters of interest using a nonparametric bootstrap that resampled households with replacement with 1,000 iterations.[5,11,32] We conducted all data management and statistical analysis in R version 3.03 (www.r-project.org).

Exploratory analyses. Following our primary analysis, we conducted a series of exploratory analyses to describe the major confounders of the relationship between poor household sanitation conditions and enteric infections. We also mapped the geographic distribution of study households and cases of enteric infection to examine the spatial relationship between sanitation conditions, population density, enteric infections, and other potentially important exposures.

We found that the composite wealth index was the single largest source of confounding for the positive associations between poor sanitation and enteric infections in Table 4. Wealth was a strong predictor of STH infection: the *A. lumbricoides* infection prevalence in the increasing four quartiles of the wealth index was 22%, 7%, 3%, and 1% (Supplemental Table 5). We observed a similar pattern of decreased infection prevalence for *E. coli* with increasing wealth, although the magnitude of gradient across wealth was less striking. Neither diarrhea nor *G. lamblia* infection exhibited the same extreme pattern of reduced infection prevalence by increasing wealth quartile (Supplemental Table 5). We observed a clear concentration of *A. lumbricoides* cases (81%) in a single village named Jumaytepeque (Supplemental Figure 1). Although similar in population density to the municipal center of Nueva Santa Rosa, *A. lumbricoides* prevalence was 9-fold higher in Jumaytepeque (27% versus 3%). Jumaytepeque also had the single largest concentration of households in the bottom quartile of the wealth index (61% in the bottom quartile), predominantly poor sanitation conditions (80% classified as poor), and abundant soil floors (56%). We did not observe this same type of extreme spatial aggregation of other enteric infections in the study population (Supplemental Figures 1–3).

Sensitivity analysis for soil-transmitted helminth detection. We had some concern that the fecal parasite concentrator assay used in the study could have low sensitivity for STH.[12] The laboratory also tested a subsample of 324 stool specimens for STH using the Kato-Katz method as part of a separate internal validation study. In a sensitivity analysis, we classified individuals as positive for STH outcomes if they were positive by either the fecal parasite concentrator assay or the Kato-Katz assay. For the 377 individuals who were only tested with the fecal parasite concentrator assay, their outcomes did not change in this analysis. The use of the composite outcome definition led to additional cases of *T. trichiura* ($N = 14$) and *A. lumbricoides* ($N = 11$) but not of hookworm. We re-estimated the association between poor sanitation and STH infection using the composite outcome definition, and the results are given in Supplemental Table 4.

REFERENCES

1. Sing T, Sander O, Beerenwinkel N, Lengauer T, 2005. ROCR: visualizing classifier performance in R. *Bioinformatics 21:* 3940–3941.
2. Hastie T, Tibshirani R, Friedman J, 2009. *The Elements of Statistical Learning*, 2nd edition. New York, NY: Springer.
3. Feiveson AH, 2002. Power by simulation. *Stata J 2:* 107–124.
4. Arnold B, Hogan D, Colford J, Hubbard A, 2011. Simulation methods to estimate design power: an overview for applied research. *BMC Med Res Methodol 11:* 94.
5. Van der Laan M, Rose S, 2011. *Targeted Learning: Causal Inference for Observational and Experimental Data*. Springer Series in Statistics. New York, NY: Springer.
6. Van der Laan MJ, Polley EC, Hubbard AE, 2007. Super learner. *Stat Appl Genet Mol Biol 6:* 25.
7. Venables WN, Ripley BD, 2002. *Modern Applied Statistics with S*, 4th edition. New York, NY: Springer.
8. Hastie T, Tibshirani R, 1990. *Generalized Additive Models*. London: Chapman and Hall.
9. Friedman J, Hastie T, Tibshirani R, 2010. Regularization paths for generalized linear models via coordinate descent. *J Stat Softw 33:* 1–22.
10. Filmer D, Pritchett LH, 2001. Estimating wealth effects without expenditure data-or tears: an application to educational enrollments in states of India. *Demography 38:* 115–132.
11. Assmann SF, Hosmer DW, Lemeshow S, Mundt KA, 1996. Confidence intervals for measures of interaction. *Epidemiology 7:* 286–290.
12. Funk AL, Boisson S, Clasen T, Ensink JHJ, 2013. Comparison of Kato-Katz, ethyl-acetate sedimentation, and Midi Parasep® in the diagnosis of hookworm, Ascaris and *Trichuris* infections in the context of an evaluation of rural sanitation in India. *Acta Trop 126:* 265–268.

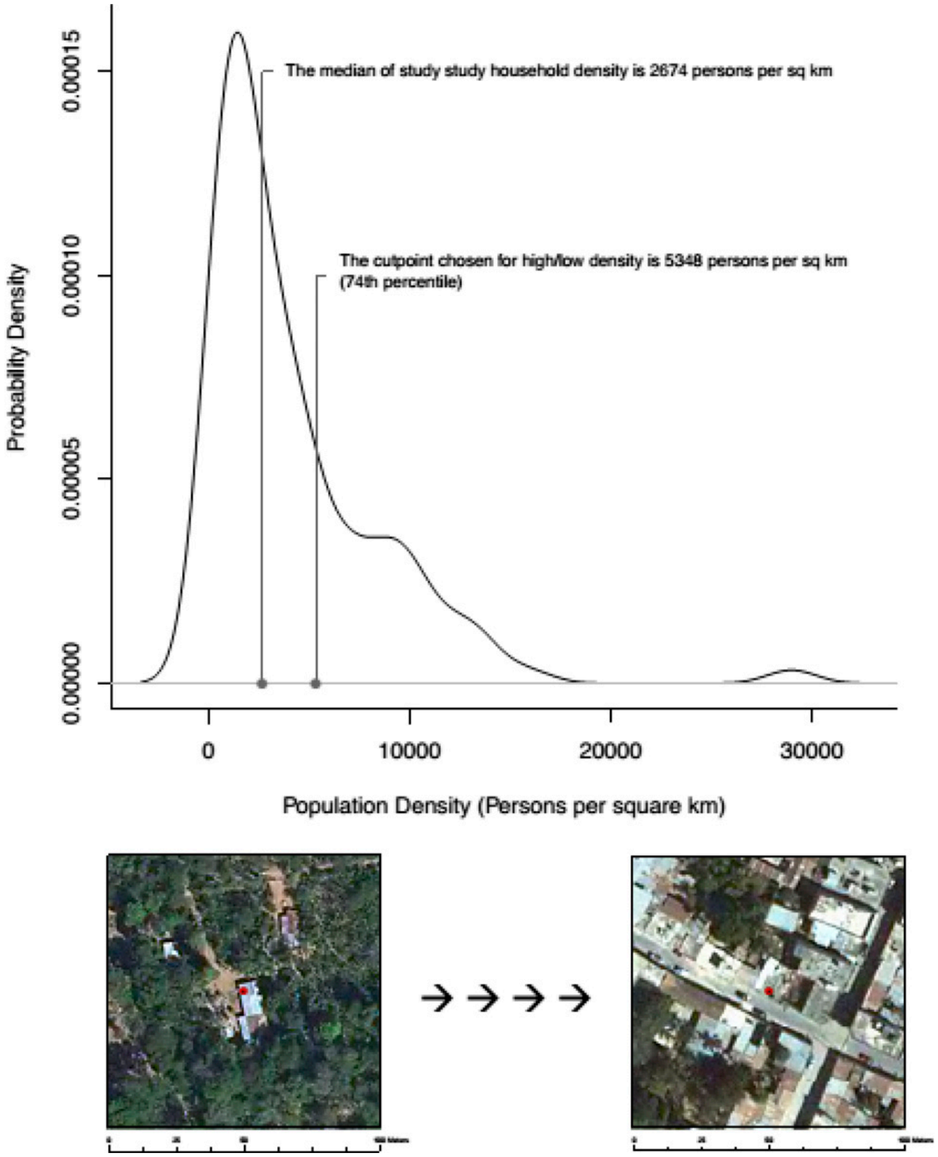

SUPPLEMENTAL FIGURE 1. Distribution of population density in the Nueva Santa Rosa Cross-sectional Survey, estimated within a 50m radius of each study household. The images show representative aerial images of study households with 100m view at the range of population density observed in the study region.

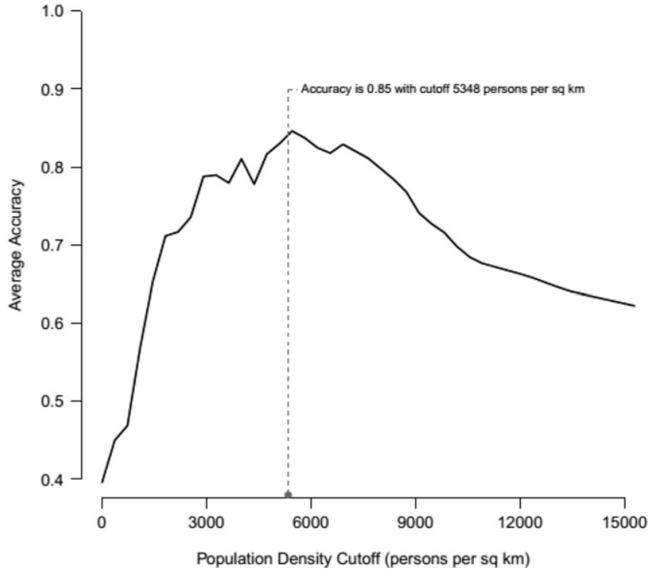

Supplemental Figure 2. Average accuracy (defined as % classification agreement) for different population density cutoffs when compared to the investigator consensus classification of "high density" and "low density" in the Nueva Santa Rosa Cross-sectional Survey.

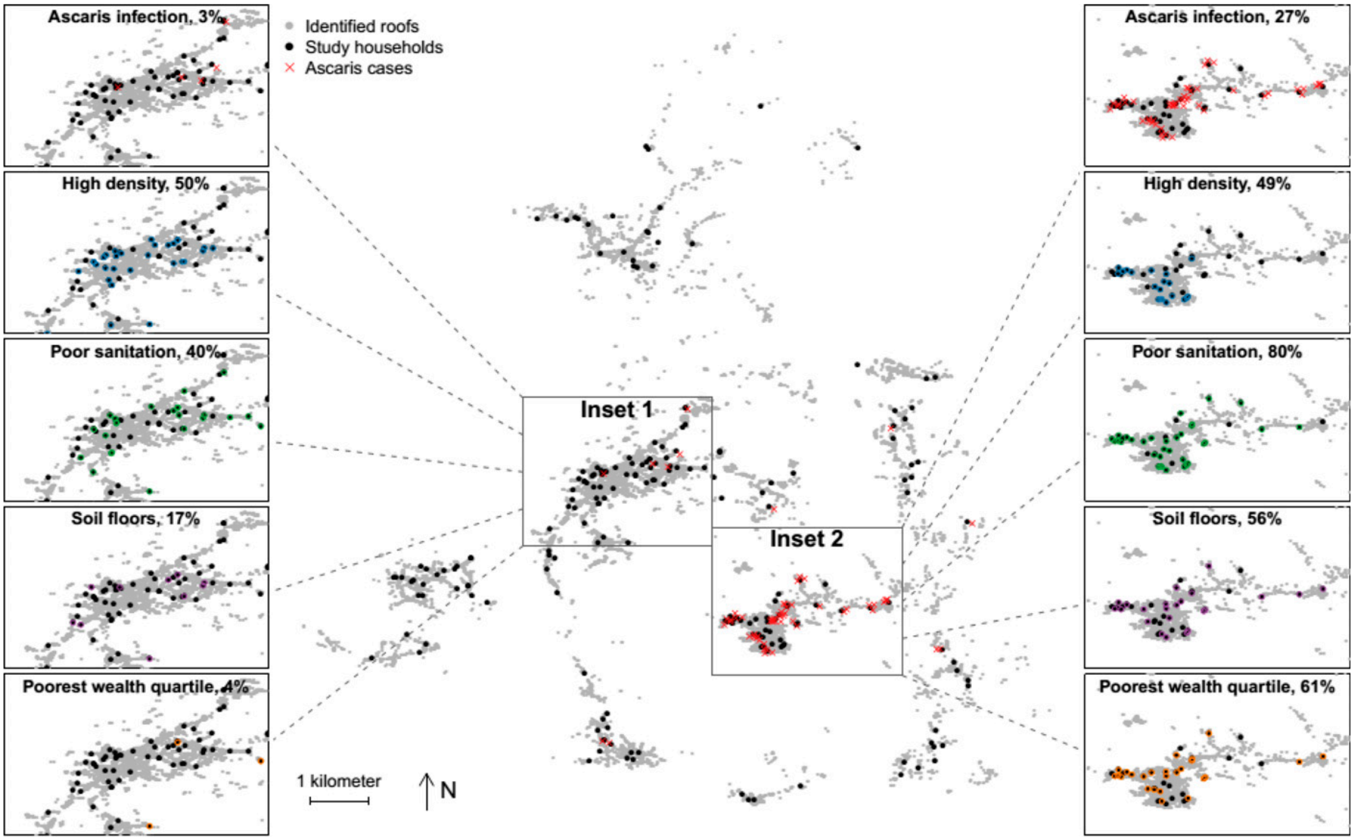

Supplemental Figure 3. Geographic distribution of 10,770 identified roofs, 204 study households, and 62 *Ascaris lumbricoides* cases in the municipality of Nueva Santa Rosa, Guatemala, 2010. Inset 1 includes the municipal center of Nueva Santa Rosa, and Inset 2 includes the town of Jumaytepeque, which differ in their environmental and wealth conditions but not in population density. The median [inter-quartile range] population density in persons per km^2 is similar for study households Inset 1 (4,966 [2,340, 9,104]) and Inset 2 (4,966 [1,528, 9,167]). Of the 62 cases of *Ascaris lumbricoides* infection in the study, 50 cases (81%) occurred in Inset 2.

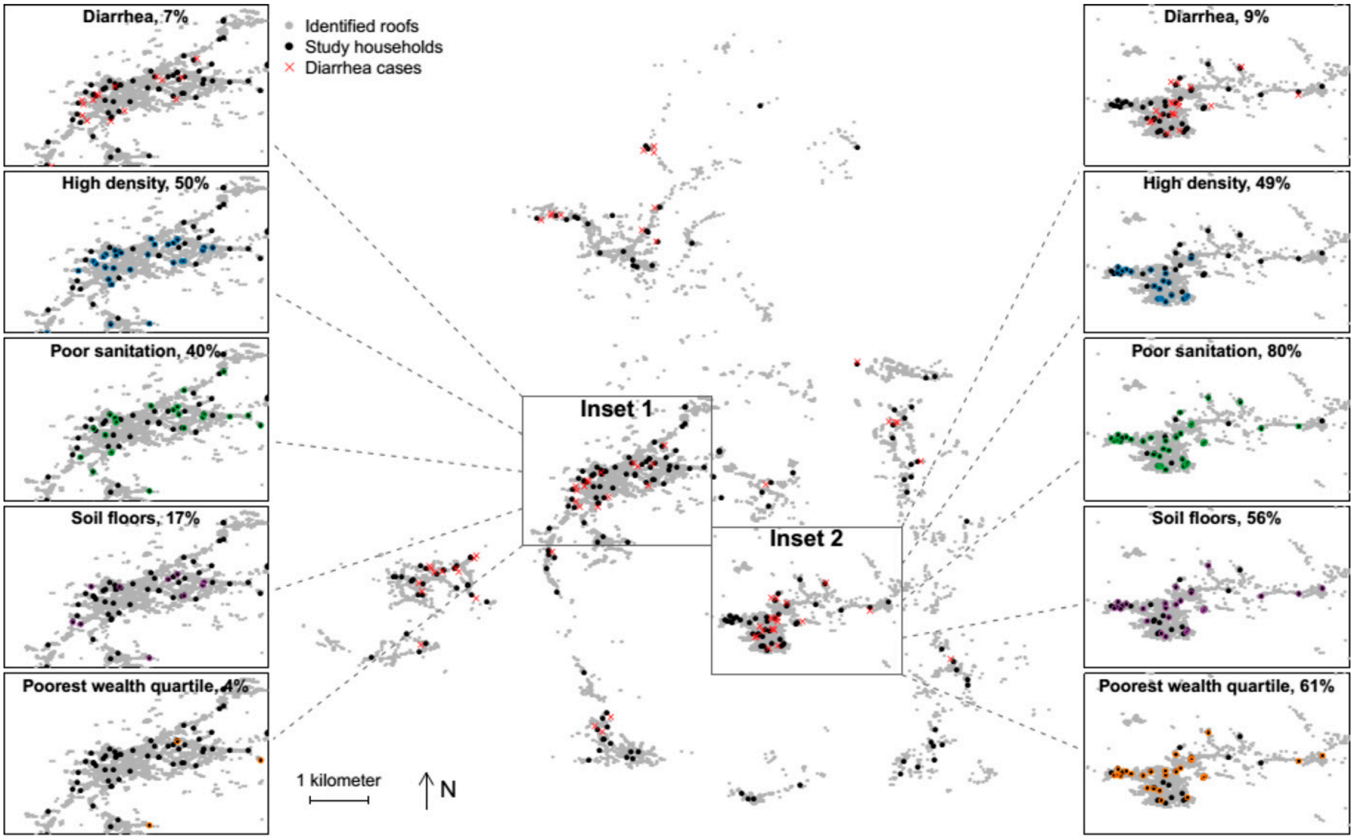

SUPPLEMENTAL FIGURE 4. Geographic distribution of 10,770 identified roofs, 204 study households, and 67 diarrhea cases in the municipality of Nueva Santa Rosa, Guatemala, 2010. Inset 1 includes the municipal center of Nueva Santa Rosa, and Inset 2 includes the town of Jumaytepeque, which differ in their environmental and wealth conditions but not in population density. The median [inter-quartile range] population density in persons per km^2 is similar for study households Inset 1 (4,966 [2,340, 9,104]) and Inset 2 (4,966 [1,528, 9,167]).

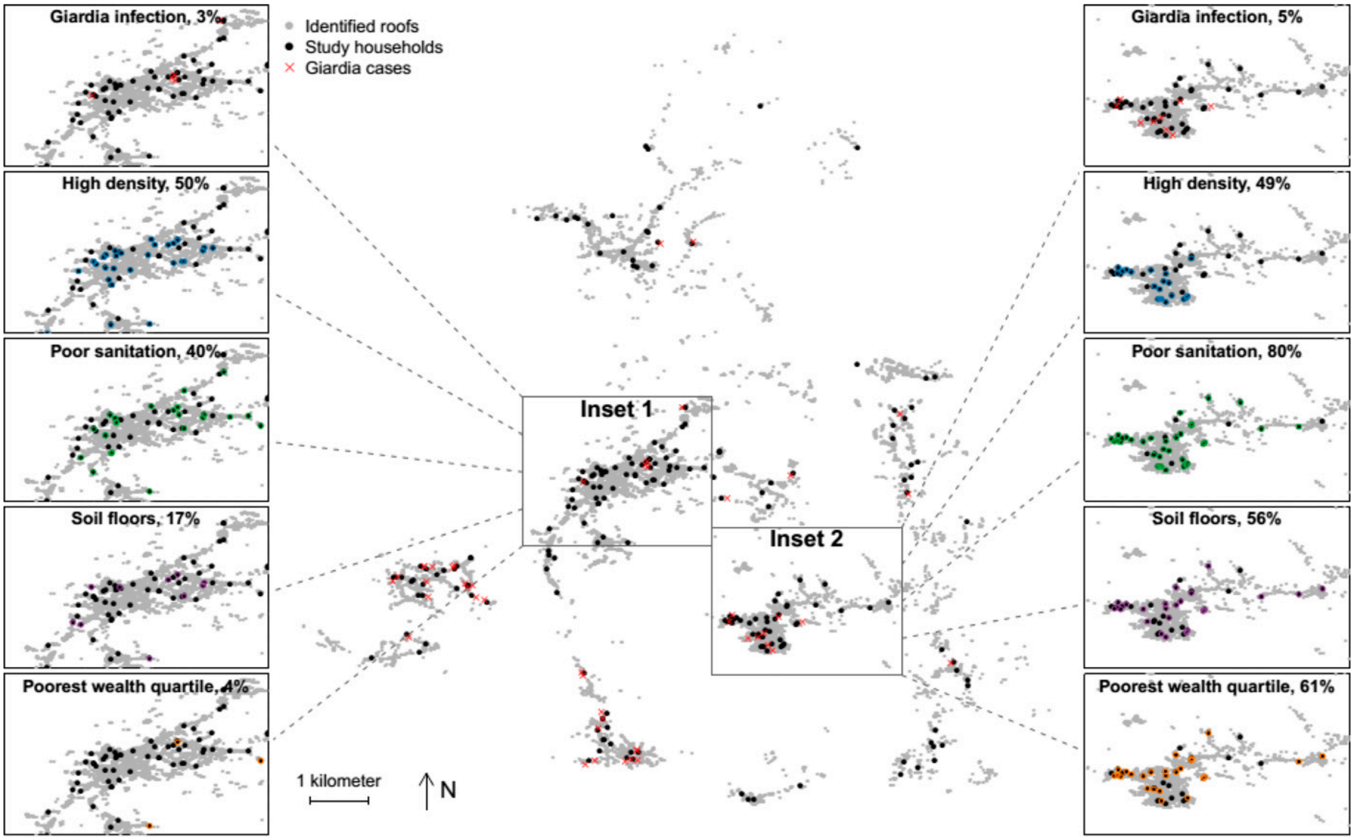

Supplemental Figure 5. Geographic distribution of 10,770 identified roofs, 204 study households, and 48 *Giardia lamblia* cases in the municipality of Nueva Santa Rosa, Guatemala, 2010. Inset 1 includes the municipal center of Nueva Santa Rosa, and Inset 2 includes the town of Jumaytepeque, which differ in their environmental and wealth conditions but not in population density. The median [inter-quartile range] population density in persons per km^2 is similar for study households Inset 1 (4,966 [2,340, 9,104]) and Inset 2 (4,966 [1,528, 9,167]).

Supplemental Table 1

Percentage of positive samples for different enteric pathogens tested in VICo surveillance diarrhea cases (Nueva Santa Rosa, 2007–2013)

Pathogen	Surveillance period	Samples tested	Positive samples	%
Bacteria				
Salmonella spp.	September 4, 2007 to December 31, 2013	3,990	8	0.2
Shigella spp.	September 4, 2007 to December 31, 2013	3,990	228	5.7
Campylobacter spp.	September 4, 2007 to December 31, 2013	3,990	271	6.8
Pathogenic *Escherichia coli**	October 31, 2007 to December 29, 2009	1,611	278	17.3
Viruses				
Rotavirus	September 4, 2007 to December 19, 2013	2,805	205	7.3
Norovirus	September 28, 2007 to December 19, 2013	2,719	335	12.3
Parasites				
Cryptosporidium spp.	September 4, 2007 to December 31, 2013	4,007	7	0.2
Entamoeba coli	September 4, 2007 to December 31, 2013	4,007	141	3.5
Giardia lamblia	September 4, 2007 to December 31, 2013	4,007	149	3.7
Ascaris lumbricoides	September 4, 2007 to December 31, 2013	4,007	151	3.8
Trichuris trichiura	September 4, 2007 to December 31, 2013	4,007	35	0.9
Hookworm	September 4, 2007 to December 31, 2013	4,007	2	0.05
Hymenolepis nana/Hymenolepis diminuta	September 4, 2007 to December 31, 2013	4,007	35	0.9

VICo = *Vigilancia Integrada Comunitaria*.

*Includes enterotoxigenic *E. coli*, enteropathogenic *E. coli*, and Shiga toxin-producing *E. coli*.

Supplemental Table 2

Age- and distance-stratified rates and IRRs for medically attended diarrhea cases in high-density vs. low-density areas of Nueva Santa Rosa, Guatemala, VICo surveillance, 2007–2013

	Low density			High density			
Outcome	Episodes	Person-years at risk	Rate*	Episodes	Person-years at risk	Rate*	High vs. low density IRR (95% CI)
Overall	1,650	83,713	197.10	2,710	111,177	243.76	1.24 (1.16, 1.32)
Age < 5 years	1,088	12,474	872.25	1,777	15,276	1163.23	1.33 (1.24, 1.44)
Age ≥ 5 years	562	71,240	78.89	933	95,900	97.29	1.23 (1.11, 1.37)
Located < 1 km	540	16,027	336.94	2,496	99,235	251.52	0.80 (0.71, 0.89)
Located ≥ 1 km	1,110	67,687	163.99	214	11,942	179.21	1.06 (0.87, 1.27)
Age < 5 years, located < 1 km	389	2,530	1537.46	1,636	13,381	1222.66	0.66 (0.59, 0.74)
Age < 5 years, located ≥ 1 km	699	9,943	702.99	141	1,896	743.71	1.16 (0.96, 1.39)
Age ≥ 5 years, located < 1 km	151	13,496	111.88	860	85,855	100.17	1.08 (0.90, 1.29)
Age ≥ 5 years, located ≥ 1 km	411	57,743	71.18	73	10,046	72.67	0.93 (0.72, 1.20)

CI = confidence interval; IRR = incidence rate ratios; VICo = *Vigilancia Integrada Comunitaria*. High-density areas were defined as populated places in the top 25% of population density for the Nueva Santa Rosa municipality.
*Incidence per 10,000 person-years.

Supplemental Table 3

Medically attended diarrhea incidence stratified by quartiles of population density and distance to surveillance sites in the municipality of Nueva Santa Rosa, VICo surveillance, 2007–2013

	Surveillance site < 1 km			Surveillance site ≥ 1 km		
Population density	Episodes	Person-years at risk	Rate*	Episodes	Person-years at risk	Rate*
Q1 (lowest)	–	–		146	12,930	112.91
Q2	540	16,027	336.94	425	23,051	184.37
Q3	–	–		513	31,705	161.80
Q4 (highest)	2,496	99,235	251.52	214	11,942	179.21

*Incidence per 10,000 person-years.

Supplemental Table 4

Sensitivity analysis of alternate *Ascaris lumbricoides* outcome definitions using FPC alone and combined with KK in the cross-sectional survey

	Improved sanitation		Poor sanitation		PR poor sanitation	Adjusted PR poor sanitation
	n/N	%	*n*/*N*	%	PR (95% CI)	PR (95% CI)
FPC						
Low density	13/212	6.1	26/260	10.0	1.63 (0.59, 4.83)	1.71 (0.61, 2.75)
High density	4/97	4.1	19/132	14.4	3.49 (1.03, 22.81)	1.33 (0.42, 3.44)
FPC + KK						
Low density	16/212	7.5	33/260	12.7	1.68 (0.76, 4.33)	1.60 (0.51, 2.26)
High density	5/97	5.2	19/132	14.4	2.79 (0.81, 25.06)	1.06 (0.35, 2.44)

CI = confidence interval; FPC = fecal parasite concentration; KK = Kato-Katz; PR = prevalence ratio. Three hundred and twenty-four of 701 participants had results from both assays, and an individual was determined positive if positive for either assay. Summary of prevalence and the PR with poor sanitation conditions, stratified by population density.

Supplemental Table 5

Enteric infection prevalence (%) by quartile of a PCA-derived wealth index in the cross-sectional survey (Nueva Santa Rosa, Guatemala, 2010)

	Quartile 1 (poorest)		Quartile 2		Quartile 3		Quartile 4 (richest)	
Outcome	*n*/*N*	% (95% CI)	*n*/*N*	% (95% CI)	*n*/*N*	% (95% CI)	*n*/*N*	% (95% CI)
Diarrhea	16/254	6.3 (2.6, 10.4)	16/235	6.8 (2.7, 11.0)	21/199	10.6 (5.8, 16.1)	13/222	5.9 (2.7, 9.5)
Ascaris lumbricoides	43/197	21.8 (12.8, 30.3)	12/181	6.6 (1.9, 12.6)	5/154	3.2 (0.8, 6.1)	1/160	0.6 (0.0, 2.4)
Giardia lamblia	10/197	5.1 (2.1, 8.2)	16/181	8.8 (5.2, 12.4)	15/154	9.7 (4.8, 15.0)	6/160	3.8 (1.1, 6.3)
Entamoeba coli	94/197	47.7 (38.7, 56.3)	56/181	30.9 (23.7, 38.9)	28/154	18.2 (11.1, 27.0)	31/160	19.4 (12.7, 26.4)

CI = confidence interval; PCA = principal component analysis. The wealth index was the first principal component from a PCA using the following household assets and income variables: refrigerator, computer, radio, clothes washer, clothes drier, car/truck, television, telephone, microwave, watch, bicycle, motorcycle/scooter, and reported household income.
